# The prechoroidal cleft in neovascular age‐related macular degeneration

**DOI:** 10.1111/aos.70035

**Published:** 2025-11-11

**Authors:** Niels J. Brouwer, T. H. Khanh Vu, Yvonne De Jong‐Hesse, Elon H. C. van Dijk

**Affiliations:** ^1^ Department of Ophthalmology Leiden University Medical Center Leiden The Netherlands; ^2^ Rotterdam Eye Hospital Rotterdam The Netherlands

**Keywords:** anti‐vascular endothelial growth factor, choroidal neovascularization, clinical significance, neovascular age‐related macular degeneration, optical coherence tomography, prechoroidal cleft, prognosis, retinal pigment epithelium detachment

## Abstract

The prechoroidal cleft is a lenticular, hypo‐reflective space on optical coherence tomography imaging, located between a band of fibrovascular material underneath the retinal pigment epithelium (RPE) and Bruch's membrane. It occurs in 8%–22% of neovascular age‐related macular degeneration (nAMD) eyes, most often with macular neovascularization (MNV) type 1 and 3, and less often with MNV type 2 or polypoidal choroidal vasculopathy. The presence of a prechoroidal cleft is associated with poor visual prognosis, some studies report more frequent occurrence of RPE tears and subretinal haemorrhages. Eyes with a prechoroidal cleft require more frequent intravitreal anti‐vascular endothelial growth factor (VEGF) injections to treat the nAMD and more often require a switch to other anti‐VEGF medication. Clinicians should be aware of the prechoroidal cleft for diagnostic and prognostic reasons, as it may be misunderstood for other (subretinal) fluid or even a choroidal lesion.

## 1 INTRODUCTION

Neovascular age‐related macular degeneration (nAMD) is a common disease in adults with a great impact on vision and quality of life (Fleckenstein et al., 2024). Intravitreal anti‐vascular endothelial growth factor (VEGF) injections are the mainstay of treatment to reduce disease activity and improve visual outcome. With the development of optical coherence tomography (OCT) imaging, and with later improvements in imaging techniques, several layers and structures of the retina and choroid have been identified that are relevant for diagnosis and follow‐up of nAMD. Examples include the presence of subretinal or intraretinal fluid (i.e. SRF and IRF, respectively), retinal pigment epithelium (RPE) detachments and hyperreflective material (at various locations in or underneath the retina) which can be related to the presence of a macular neovascularization (MNV). MNV's—formerly termed choroidal neovascularization (CNV)—occur in different types, with a prevailing classification into MNV type 1, 2 and 3, and the associated polypoidal choroidal vasculopathy (PCV) which is often seen as a subtype of MNV type 1 (Spaide et al., 2020).

About a decade ago, a new feature on OCT imaging was reported in nAMD (Mukai et al., 2014; Nagiel et al., 2013): a lenticular hypo‐reflective space, between Bruch's membrane and the RPE, with a band of hyperreflective (fibrovascular) material near the RPE. This was termed a ‘cleft’ and later a ‘prechoroidal cleft’. The finding followed the ‘triple layer sign’ that was reported in patients with PCV in 2012 (Khan et al., 2012), identifying a hypo‐reflective space underneath the neovascular and PCV tissue, between a detached Bruch's membrane and the choroid. This triple layer sign was seen by the authors as a support to relate PCV to neovascular tissue rather than to choroidal vasculature. In the AMD studies, the prechoroidal cleft was soon linked to prognosis of the disease, as it was linked to a higher risk of RPE tearing following anti‐VEGF treatment (Nagiel et al., 2013). Later reports in nAMD had different findings regarding prognostic and clinical implications of this prechoroidal cleft, and it is only in recent years that a few larger series on its occurrence have been reported.

Recognition of the prechoroidal cleft in nAMD is important for clinicians as it may be confused with other (subretinal) fluid or even choroidal lesions and has a prognostic implication for patients. In this work, we review and interpret the current literature on the prechoroidal cleft with recommendations for clinical practice.

## 2 METHODS OF LITERATURE SEARCH

We performed a literature search using databases of PubMed, MEDLINE, Web of Science and Google, using search terms as ‘prechoroidal cleft’, ‘neovascular age‐related macular degeneration’, ‘choroidal neovascularization’, ‘triple layer sign’ and ‘(multi‐layered) pigment epithelial detachment’. We included original works (i.e. case reports, cases series, cohort studies, and trials), published in peer‐reviewed journals, with availability of full text in English, available up to April 2025. Papers had to address nAMD patients, report on the presence of a prechoroidal cleft (or synonymous terminology) and present clinical patient data. Review articles were excluded, but—together with the other papers—bibliographies were further evaluated to find additional relevant works. Papers were manually reviewed, and no artificial intelligence was used to screen or identify papers. Risk of bias was assessed for each study in the context of retrieved data, taking in account the selection of patients, received treatments and reported outcome measures. Quality was assured by only selecting work from peer‐reviewed journals. The search (as depicted in the PRISMA‐style flow chart, see Figure S1) led to an overview of available literature on the prechoroidal cleft, presented in Table 1.

**TABLE 1:** Literature on the prechoroidal cleft in neovascular age‐related macular degeneration (nAMD).

Study	Study type^a^	Origin	Patients	Size	Incidence of a PC^d^	Prognostic associations with a PC	Baseline findings
Forte et al. (2024)	Case report	Italy	nAMD	1	N/A	PC regression after switch to brolucizumab	
Sariyeva Ismayilov et al. (2024)	Retrospective case–control	Turkey	nAMD	136	4/136 = 2.9%	PC not related to SRF resolution	No baseline data
Kredi et al. (2023)	Retrospective case–control	Israel	nAMD	140	21/140 = 15%	PC required 1.29 times more injections with anti‐VEGF and more often a medication switch. PC had more often SRF during follow‐up. BCVA was equal at follow‐up. PC was not related to MNV type. Complications not mentioned.	Gender/age not different. PC group had less often IRF at baseline BCVA equal at baseline
Yeom et al. (2023)	Retrospective case–control	Korea	nAMD (refractory)	81	26/81 = 32.1%	PC relates to good response to brolucizumab	No baseline data
Cozzi et al. (2022)	Retrospective case series	Italy	nAMD with PC	27	N/A	Increase in PC size with macular neovascular reactivation and reduction of size after treatment. PC most with MNV type 1, then type 3 and then mixed 1–2.	BCVA not related to PC size No data on SRF/IRF
Hayashi‐Mercado et al. (2022)	Retrospective case–control	Mexico	nAMD with MNV type 1–2	83	18/83 = 22%	PC is unfavourable for vision gain after treatment	PC more often in females (30%) than males (11%); *p* = 0.04. Age not different. No data for SRF/IRF
Kim et al. (2022)	Retrospective case series^b^	Korea	nAMD with PC	63	N/A	Over time, mean PC thickness decreased in the cohort. Most pt. had MNV type 1, then MNV type 2 and then MNV type 3 PC thickness related to fibrotic scar formation at 12 months	No comparative data on gender/age, or SRF/IRF
Kim et al. (2021)	Retrospective case series^c^	Korea	nAMD (incl PCV)	490	Total: 61/490 = 12% Only AMD: 51/253 = 20.2% Only PCV: 10/237 = 4.2%	PC regressed in 28%; regression is favourable with visual prognosis Most PC had MNV type 3, then MNV type 1 and then MNV type 2	Gender/age not different No comparative data on SRF/IRF
Pece et al. (2019)	Case report	Italy	nAMD	1	N/A	Remarkably shaped PC	
Kim et al. (2018)	Retrospective case–control	Korea	nAMD with MNV type 3	166	37/166 = 22.3%	BCVA at final visit worse in the PC group PC group required 1.39 times more anti‐VEGF injections. PC group more often RPE tear and subretinal haemorrhage No relation with MNV types presented: all MNV type 3	Gender/age not different. No data on SRF/IRF BCVA equal at baseline
Kim et al. (2017)	Retrospective case–control	Korea	nAMD	234	Total: 29/234 = 8.1% Only nAMD+RAP: 22/106 = 21% Only PCV: 7/128 = 5.5%	BCVA at final visit worse for PC (especially if early event). PC group more often MNV type 1 or 3, less often MNV type 2 PC group 1.40 times more anti‐VEGF injections and less often complete response PC group more often submacular haemorrhage treated with intravitreal gas (*p* = 0.003).	PC group older (73 vs. 68y). Gender not different No data on SRF/IRF BCVA equal at baseline
Mukai et al. (2014)	Retrospective case–control	Japan	nAMD	227	Total: 22/227 = 10% Only nAMD: 14/105 = 13% Only nAMD+RAP: 17/118 = 14% Only PCV: 5/109 = 5%	PC most often with MNV type 1 and 3; least in type 2 or PCV No comparative prognostic/baseline data PC resolved in 14/22 pt In 5/22 pt. = 23% with cleft, a RPE tear developed	No data on age Gender not different.
Rahimy et al. (2014)	Retrospective case series	USA / Denmark	nAMD, with multilayered PED	34	N/A	In 34 pt. with a multilayered PED, 25 had a PC	
Nagiel et al. (2013)	Retrospective case series	USA / Denmark	nAMD, with RPE tear	8	N/A	6/8 pt. had an outward bowing of Bruch's membrane.	
Khan et al. (2012)	Retrospective case series	USA	PCV, with various disorders	18	N/A	4/18 pt. with PCV had a ‘triple layer sign’	

Abbreviations: BCVA, best‐corrected visual acuity; IRF, intraretinal fluid; MNV, macular neovascularization; N/A, not applicable; nAMD, neovascular age‐related macular degeneration; PC, prechoroidal cleft; PCV, polypoidal choroidal vasculopathy; PED, retinal pigment epithelium detachment; RAP, retinal angiomatous proliferation; RPE, retinal pigment epithelium; SRF, subretinal fluid.

^a^ For the purpose of this work, studies were deemed ‘case–control’ if a comparison between patients with and without a prechoroidal cleft is reported, otherwise, the study was deemed ‘case series’ in the absence of comparative data for a PC. This can differ from the study type as mentioned by the authors.

^b^ In this work, patients with and without a specific retinal layer (‘layer 2’) were compared, not with and without a prechoroidal cleft.

^c^ In this work, patients with AMD were compared to patients with PCV.

^d^ Incidence rates are presented for the study group as a whole, and if applicable for subgroups as well.

## 3 RESULTS AND DISCUSSION

### 3.1 Features of the prechoroidal cleft

**FIGURE 1:**
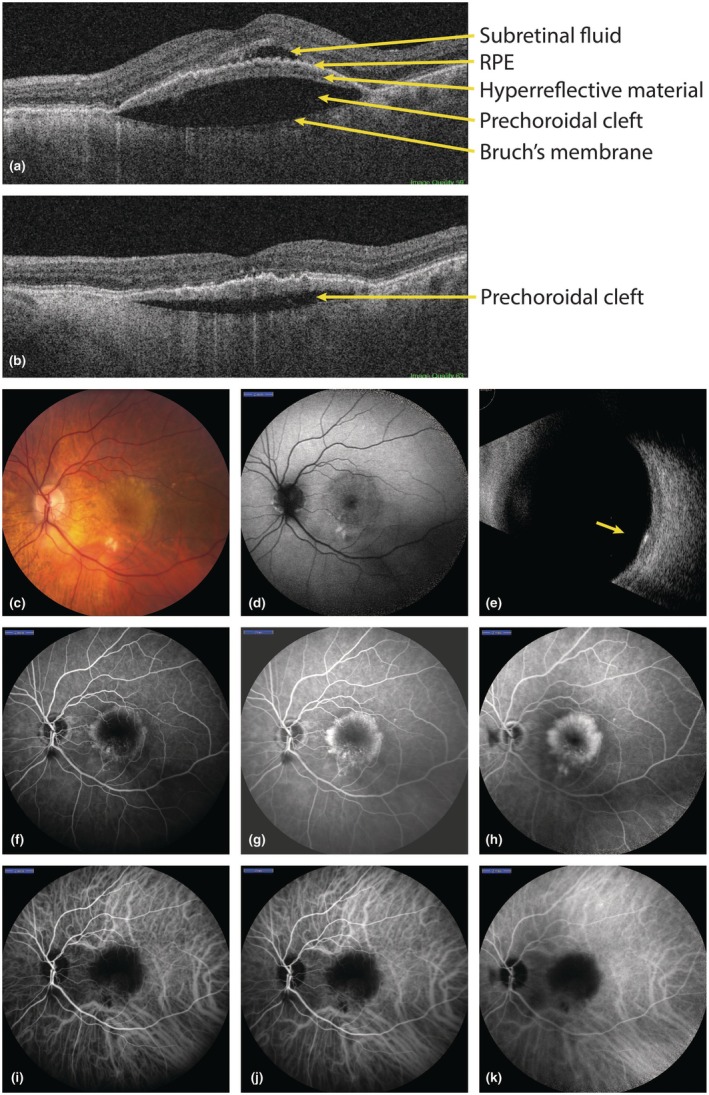
Clinical presentation of a large prechoroidal cleft in a 80‐year‐old patient who was under anti‐vascular endothelial growth factor (VEGF) therapy for neovascular age‐related macular degeneration (nAMD). (a) The prechoroidal cleft is located between Bruch's membrane and a band of hyperreflective material underneath the retinal pigment epithelium (RPE). Note the subretinal fluid accompanying this case, indicative of active disease. (b) Same patient, 5 months later after treatment with anti‐VEGF injections and resolution of the subretinal fluid, and regression of the prechoroidal cleft. (c) Fundoscopy demonstrating the nAMD in this patient at the time of image A, with an extensive amount of drusen. (d) Fundus autofluorescence demonstrating a hypo‐autofluorescent fovea. (e) Ultrasound imaging showing a mid‐reflective retinal lesion (arrow), and the absence of choroidal lesions or pathology. (f–h) Fluorescein angiography showing central leakage (image F at 1 min, G at 3 min, H at 10 min). (i–k) Indocyanine green angiography showing central blockage (image i at 1 min, j at 3 min, k at 10 min).

The prechoroidal cleft is a sign on OCT in nAMD eyes (Figure 1). In current literature, three components are used to define it, being a (1) hypo‐reflective space, (2) lenticular shaped and (3) located between Bruch's membrane and a fibrovascular band underneath the RPE (Hayashi‐Mercado et al., 2022; Kim et al., 2017, 2018, 2021; Kredi et al., 2023; Mukai et al., 2014; Nagiel et al., 2013). The hyporeflective space is assumed to be filled with fluid, with similar reflectivity as SRF or a serous RPE detachment, although the exact nature or components are unknown in the absence of studies on histology (Mukai et al., 2014). The lenticular shape is formed by an ‘outward bowing’ towards the choroid (more extensively than the natural curve of the eye), to differentiate it from a regular RPE detachment. The prechoroidal cleft is located between Bruch's membrane and the RPE, with fibrovascular (hyperreflective) tissue at the inner RPE side (resulting in what some authors call a ‘multi‐layered pigment epithelium detachment’; Rahimy et al., 2014), and Bruch's membrane on the outward side of the cleft. To recall, this is slightly different from the ‘triple layer sign’ by Khan et al. (2012) that assumed a hypo‐reflective space between the choroid and a Bruch's membrane that was attached to the undersurface of a PCV lesion.

Larger prechoroidal clefts can be seen with fundoscopy as an elevated area of the retina (Figure 1). Fluorescein angiography may demonstrate leakage of retinal vessels (and early hyperfluorescence), similar to what is seen in serous RPE detachments (Mrejen et al., 2013). It is not known, however, to what extent the cleft influences the angiographic image of the detachment itself. Indocyanine green angiography may demonstrate blockage due to the tissue overlying the choroid and the cleft obstructing the choroidal vessels, but as with fluorescein angiography the exact depiction of the cleft in addition to the RPE detachment is not known. Ultrasound imaging has no particular role in demonstrating the presence of a prechoroidal cleft but helps to exclude the presence of choroidal pathology or lesions that may mimic the elevated mass on fundoscopy.

### 3.2 Incidence

#### 3.2.1 Total incidence in nAMD

In studies on nAMD, a prechoroidal cleft is reported in 8–22% of patients (Table 1). The wide range most likely results from the varying included subtypes of AMD, as the presence is more abundant in classic nAMD (with or without retinal angiomatous proliferation) with incidences of 20%–21%, than it is in PCV with incidences of 4%–5% (Kim et al., 2017, 2021). The occurrence of subtypes of AMD varies between populations of different ethnic backgrounds, calling for caution when analysing the crude incidence rates of different studies. In most Asian cohorts, the incidence of a prechoroidal cleft is below or at 10% (Kim et al., 2017, 2021; Mukai et al., 2014), while in a large Caucasian cohort (with fewer PCV cases), the incidence was 15% (Kredi et al., 2023), and in a Hispanic cohort (lacking PCV at all), it was 22% (Hayashi‐Mercado et al., 2022). Excluding cases with PCV from the Asian reports, however, the incidence of a prechoroidal cleft is comparable to the other works with 13%–22% (Kim et al., 2017, 2018, 2021; Mukai et al., 2014).

#### 3.2.2 Timing of development

The occurrence of a prechoroidal cleft does not necessarily concur in time with the diagnosis of nAMD. The mean time between diagnosis of an MNV and the detection of a prechoroidal cleft was 15.4 months in a large cohort (Kim et al., 2021), which may be due to time needed for the cleft to develop following hydrostatic changes from the MNV. This will be further discussed in Section 3.6 of this paper. The wide spread of reported incidences of a prechoroidal cleft may therefore also relate to different follow‐up points at which the presence was assessed. In specific studies, this results in 2.9% of treatment‐naïve nAMD (Sariyeva Ismayilov et al., 2024), up to 32.1% of refractory nAMD following several cycles of anti‐VEGF injections (Yeom et al., 2023). Again, this emphasizes that selection bias may occur with comparison of crude rates.

### 3.3 Baseline relations

#### 3.3.1 Age/gender

One study reports that nAMD patients with a prechoroidal cleft were older than those without (mean age of 73 years vs. 68 years (*p* = 0.01), respectively) (Kim et al., 2017), but most studies find no age difference (Hayashi‐Mercado et al., 2022; Kim et al., 2018, 2021; Kredi et al., 2023). Similarly for gender, one study reports that a prechoroidal cleft was seen more often in female compared to male patients (occurring in 30% vs. 11% (*p* = 0.04), respectively) (Hayashi‐Mercado et al., 2022), but most works show a statistically equal distribution (Kim et al., 2017, 2018, 2021; Kredi et al., 2023).

#### 3.3.2 SRF and IRF

Presence of SRF did not differ based on the presence of a prechoroidal cleft in nAMD patients, but in the presence of a prechoroidal cleft, less often IRF was observed at the start of treatment (in 39% vs. 70%; *p* = 0.016) (Kredi et al., 2023). However, the fact that most studies do not present data on these relative risks limits the strength of this association and clinical relevance.

#### 3.3.3 MNV types

The occurrence of a prechoroidal cleft in nAMD may be related to certain types of MNV. A prechoroidal cleft is seen most often with MNV type 1 or MNV type 3, and less often with MNV type 2 or PCV (Cozzi et al., 2022; Kim et al., 2017, 2021; Mukai et al., 2014). In a composite of four studies, this results in the occurrence of a prechoroidal cleft in 12.5% of patients with MNV type 1, in 8.0% of MNV type 2, in 27.5% of MNV type 3 and in 4.6% of PCV (Table 2). Interestingly, in a Caucasian cohort of 140 patients, a high number of MNV type 2 was found and no statistically significant relation was found for the presence of a prechoroidal cleft and a specific MNV subtype (Kredi et al., 2023). These two findings differ from the other works in which mainly patients with an Asian ethnicity were included.

**TABLE 2:** Occurrence of a prechoroidal cleft with different macular neovascularization types.

Study	MNV type 1^a^	MNV type 2^a^	MNV type 3^a^	PCV^a^
Kredi et al. (2023)	5/48 = 10%	10/57 = 18%	0/11 = 0%	0/1 = 0%
Kim et al. (2017)	17/120 = 14%	3/82 = 4%	9/32 = 28%	7/128 = 5.5%
Mukai et al. (2014)	12/67 = 18%	2/38 = 5%	3/13 = 23%	5/109 = 5%
Kim et al. (2021)	33/302 = 11%	10/135 = 7%	18/53 = 34%	10/237 = 4.2%
Composite	67/537 = 12.5%	25/312 = 8.0%	30/109 = 27.5%	22/474 = 4.6%

Abbreviations: MNV, macular neovascularization; PCV, polypoidal choroidal vasculopathy.

^a^ Data from four studies was used to determine a composite rate of occurrence. Rates presented as ‘patients with prechoroidal cleft’/‘total study size’ = ‘% of patients with prechoroidal cleft’.

### 3.4 Prognostic influence

#### 3.4.1 Visual acuity

At baseline in the reported studies, the presence of a prechoroidal cleft was not related to visual acuity (Cozzi et al., 2022; Kim et al., 2017, 2018; Kredi et al., 2023). It was hypothesized that this may be because the photoreceptors are located above the RPE and are not directly influenced by the prechoroidal cleft (Cozzi et al., 2022). However, the visual prognosis is different when patients are followed over time and after treatment with anti‐VEGF injections. The presence of a prechoroidal cleft was unfavourable for visual gain after treatment, which was suggested to be related to subretinal haemorrhage and RPE tear formation as will be discussed later on (Hayashi‐Mercado et al., 2022; Kim et al., 2018). Mostly, visual prognosis was worse if the prechoroidal cleft had an early onset after diagnosis, defined as development within 6 months after diagnosis of nAMD (Kim et al., 2017). The authors suggest that those patients may have had a poor response to anti‐VEGF treatment in the initial phase already, or more fibrosis development early on. Further, regression of the prechoroidal cleft during treatment gives a more favourable visual prognosis, which was related to less fibrotic scarring and apparent photoreceptor preservation (Kim et al., 2021).

#### 3.4.2 Complications

**FIGURE 2:**
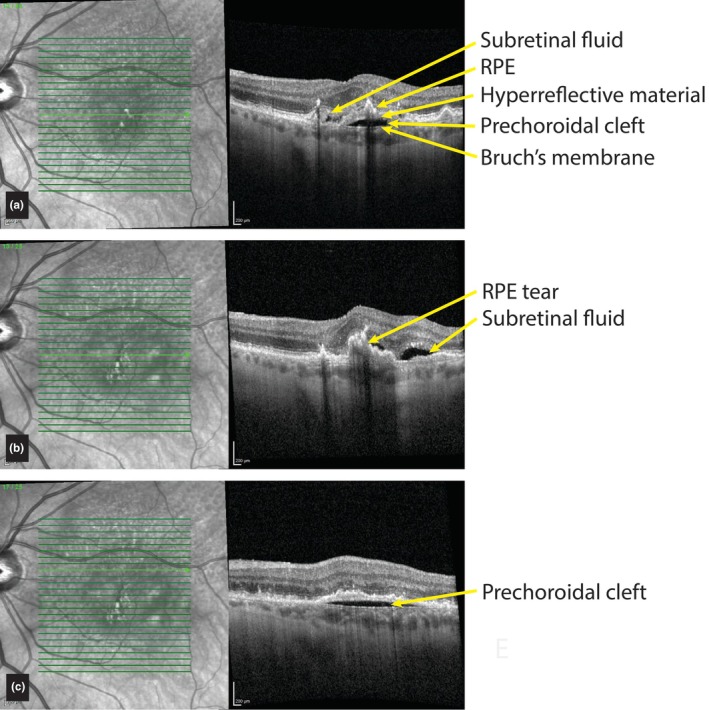
Prechoroidal cleft and retinal pigment epithelium (RPE) tear. (a) This 79‐year‐old patient was followed for several years with dry age‐related macular degeneration and developed mild subretinal fluid and a flat RPE detachment with a small prechoroidal cleft, for which anti‐vascular endothelial growth factor (VEGF) injections were started. (b) Three months later, an RPE tear developed with an increase of subretinal fluid, requiring a switch to another type of anti‐VEGF medication. (c) At the time of RPE tear development, a prechoroidal cleft was noticed slightly superior to the fovea. This disappeared after 3 months and reappeared 8 months later. While receiving anti‐VEGF therapy, this cleft remained in the years to follow.

**FIGURE 3:**
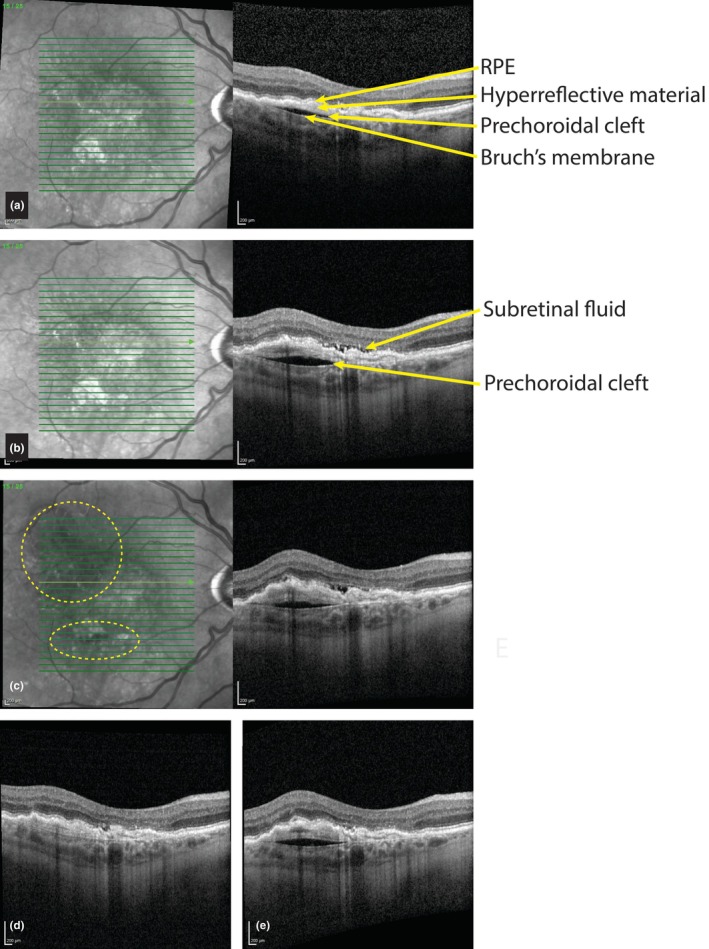
Prechoroidal cleft and retinal haemorrhage. (a) This 78‐year‐old patient was treated with anti‐vascular endothelial growth factor (VEGF) injections for neovascular age‐related macular degeneration (nAMD) for many years. A prechoroidal cleft developed in the absence of subretinal or intraretinal fluid. (b) Six months later, mild subretinal fluid developed for which the interval of anti‐VEGF injections was shortened. (c) Two months later a retinal haemorrhage developed (best seen en face within the dotted circles). (d) Again 2 months later, both the subretinal fluid and cleft had resolved. (e) After 4 months, while still receiving anti‐VEGF injections every 6 weeks, the prechoroidal cleft re‐appeared.

**FIGURE 4:**
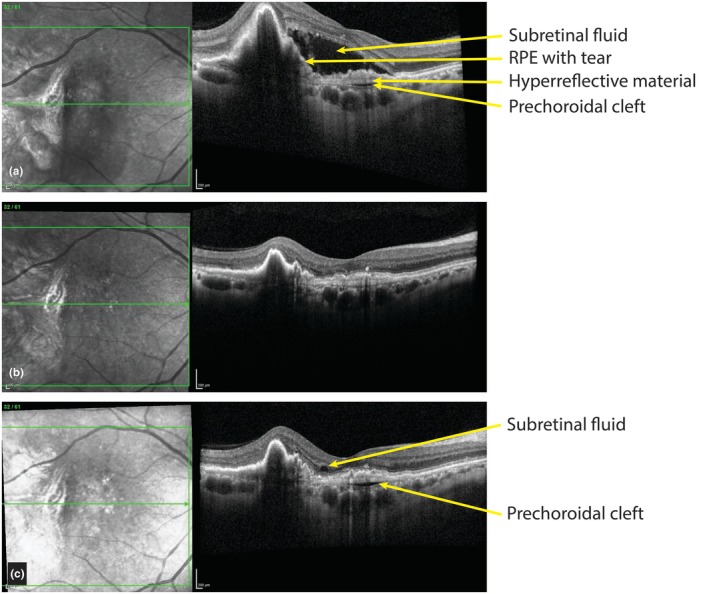
A small prechoroidal cleft with a large retinal pigment epithelium (RPE) tear. (a) This previously untreated 63‐year‐old patient developed blurry vision in the right eye and was diagnosed with neovascular age‐related macular degeneration with subretinal fluid and a large RPE tear. A small prechoroidal cleft can be seen nasally from the tear. Anti‐vascular endothelial growth factor injections were started. (b) Eight months later, the subretinal fluid and prechoroidal cleft had both resolved. (c) Four months later, minor subretinal fluid and a small prechoroidal cleft re‐appeared.

In earlier work, the presence of a prechoroidal cleft in nAMD was linked to more frequent occurrence of RPE tears and subretinal haemorrhages (Figures 2, 3, 4) (Kim et al., 2018; Mukai et al., 2014; Nagiel et al., 2013). Mukai reported a RPE tear in 5/22 (23%) of patients with a prechoroidal cleft, but did not compare this to a reference group without a cleft (Mukai et al., 2014). Later work by Kim et al. (2017) found an equal occurrence of subretinal haemorrhage in the presence or absence of a cleft, but found that haemorrhage with vision deterioration (requiring intravitreal gas injection), was seen 3 times more in the prechoroidal cleft patients compared to those without (*p* = 0.003) (Kim et al., 2017). Kim et al. (2018) reported a RPE tear in 8/37 patients (22%; vs. 4% in controls, *p* = 0.002) and subretinal haemorrhage in 10/37 patients (27%; vs. 5% in controls, *p* < 0.001) (Kim et al., 2018). Not all studies report the association between the prechoroidal cleft and either RPE tears or subretinal haemorrhages however (Cozzi et al., 2022; Kim et al., 2021; Kredi et al., 2023), suggesting a trend that warrants further research.

Another complication that has been mentioned with prechoroidal cleft presence was the development of fibrotic scarring. One study reported that fibrotic scar formation at 12 months was related to thickness of the cleft (*p* = 0.038) (Kim et al., 2022). In multivariate regression this relation lost statistical significance however, and other factors (as the presence of an additional ‘layer 2’ in the multilayered pigment epithelium detachment) were of more importance. Fibrotic scar formation is multifactorial and may occur despite anti‐VEGF therapy (Tenbrock et al., 2022), questioning a true link with a prechoroidal cleft, or perhaps suggesting a common inflammatory background.

#### 3.4.3 Disease activity

Some authors relate the presence of a prechoroidal cleft to nAMD activity (Mukai et al., 2014), and present it as a potential biomarker (Cozzi et al., 2022). Cozzi et al. (2022) found that prechoroidal cleft size related to macular neovascular activity, and the cleft decreased in size after the initiation of anti‐VEGF treatment. Kredi et al. (2023) found that SRF (as indicator of active disease) was more likely present at 12 months (65% vs. 28%, p = 0.002) and 24 months (47% vs. 20%, *p* = 0.018) in eyes with a prechoroidal cleft compared to eyes without. The value as a biomarker in addition to other OCT findings is not yet clear, however.

### 3.5 Treatment consequences

#### 3.5.1 Anti‐VEGF injections

The presence of a prechoroidal cleft has implications for the treatment of the associated nAMD. In eyes with a prechoroidal cleft, following regular pro‐re‐nata and treat and extend regimens, more injections with anti‐VEGF were required, and more often a switch to other types of anti‐VEGF was needed to resolve the SRF or IRF of the nAMD (Kim et al., 2017, 2018; Kredi et al., 2023). From studies that report the number of injections, it can be calculated that eyes with a prechoroidal cleft received a mean of 1.29 (Kredi et al., 2023), 1.39 (Kim et al., 2018) or 1.40 times (Kim et al., 2017) the amount of injections that was given to non‐prechoroidal cleft eyes. This is a remarkably similar figure for the three studies. Despite these frequent injections, however, less often a complete response (i.e. disappearance of fluid of the nAMD, not of the prechoroidal cleft itself) was reached (Kim et al., 2017).

Interestingly, new anti‐VEGF agents may be more effective in nAMD with a prechoroidal cleft. In one study after initial treatment with bevacizumab no relation was found between SRF resolution and prechoroidal cleft presence (Sariyeva Ismayilov et al., 2024). Another work in refractory nAMD cases, with failure to previous anti VEGF therapy and a switch to brolucizumab, showed that presence of a prechoroidal cleft related to good therapy response (Yeom et al., 2023). This comparison is prone to bias however, with different patient selection and follow‐up, but may instigate future works.

#### 3.5.2 Will the prechoroidal cleft resolve?

Treatment of the prechoroidal cleft itself has—to our knowledge—not been subject of debate, and no treatment is suggested in the literature. Commonly a prechoroidal cleft will resolve or regress in size, but this may take several months or years. At the final visit in their studies, the prechoroidal cleft had regressed in 17/61 (28%) patients (mean follow‐up time 46.7 months) (Kim et al., 2021), 9/21 (43%) patients (mean follow‐up time 34 months) (Kredi et al., 2023) and 14/22 (64%) patients (no follow‐up time specified) (Mukai et al., 2014). Measuring the size of a prechoroidal cleft, there was a reduction after start of anti‐VEGF therapy (Cozzi et al., 2022; Kim et al., 2022). Not all studies report the presence of prechoroidal clefts during follow‐up, however (Kim et al., 2017, 2018). Fluctuating over time, a prechoroidal cleft may also disappear and reappear (Figure 3). In one study after resolution in 6/21 (29%) patients under anti‐VEGF therapy, it reappeared in three cases (Kredi et al., 2023).

**FIGURE 5:**
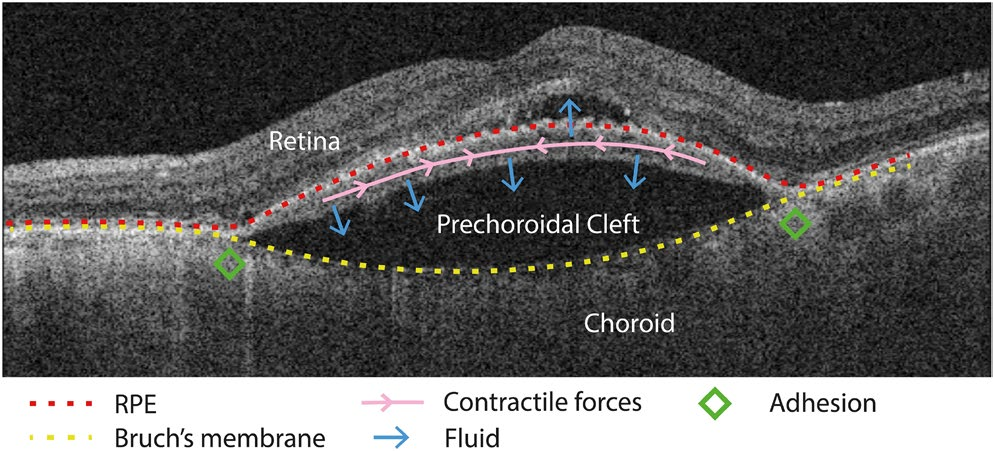
Mechanisms of prechoroidal cleft development. Postulated mechanisms for the prechoroidal cleft to develop: Hydrostatic pressure from the neovascular material, in conjunction with contractile forces of this material, causes the cleft to expand towards the choroid. Tight adhesions between Bruch's membrane and the retinal pigment epithelium (RPE) delineate the cleft, whereas loose adhesions allow for separation of the layers.

### 3.6 Mechanisms of development

Several authors speculated on the mechanism of origin of the prechoroidal cleft. It is important to note that these theories have been based on imaging techniques only, since no reports on histopathology regarding a cleft are known (Kim et al., 2021).

Most authors describe two driving forces for the prechoroidal cleft to develop, as was already postulated early on by Nagiel et al. (2013) (Figure 5). There is hydrostatic pressure from fluid leaking from the MNV material, and contractile forces from the MNV material that support the outward bowing of Bruch's membrane. In actively leaking MNV's, fluid may thus leak anteriorly towards the retina (causing IRF and SRF), and posteriorly towards Bruch's membrane (causing the cleft to develop) (Mukai et al., 2014). This would support the view that a prechoroidal cleft is a sign of active disease and MNV activity (Cozzi et al., 2022; Mukai et al., 2014). It fits the observations that better regulation of the MNV with proper anti‐VEGF therapy (thereby limiting leakage) also causes the prechoroidal cleft to regress.

Contraction of the MNV material is the second proposed driving force of a prechoroidal cleft. This may be either spontaneously or due to therapy (Mukai et al., 2014). It is hypothesized that sub‐RPE tissue and Bruch's membrane attach weak and firm in various locations (Kim et al., 2018; Nagiel et al., 2013). When MNV material contracts, the weak attachments allow for outward bowing of Bruch's membrane, resulting in a prechoroidal cleft with firmer attachments at the border of the cleft (Figure 5). In ultimo, the MNV contraction may even cause RPE tears (Nagiel et al., 2013) and rupture of vessels located at the firmly‐attached areas (Kim et al., 2018). This would explain the higher observed frequency of subretinal haemorrhage and RPE tears in eyes with a prechoroidal cleft.

The association between prechoroidal clefts and different types of MNV calls for an understanding of the different MNV types. The following terminology prevails (Spaide et al., 2020): *MNV type 1 (formerly CNV type 1, ‘occult’)* is a vascular ingrowth from the choriocapillaris into the sub‐RPE space. *MNV type 2 (formerly CNV type 2, ‘classic’)* is a vascular ingrowth from the choroid into the subretinal space (i.e. above the RPE). *MNV type 3 (formerly CNV type 3, ‘retinal angiomatous proliferation’)* is a vascular growth from the deep capillary plexus of the retina into the outer retina layers or RPE (i.e. not necessarily involving the choroid). *PCV* consists of a branching network with aneurysmal dilatations, in the sub‐RPE space at the edge of a vascular lesion, and is now often seen as a subtype of MNV type 1. Notably, PCV can occur with AMD but also with other diseases on the pachychoroid spectrum (Sirks et al., 2025). Since type 1 and 3 MNV grow into (or near) the sub‐RPE space, at the exact location of postulated weak adhesions needed for the prechoroidal cleft, this may explain why these clefts are seen more frequently with these types of MNV than with type 2 MNV (that remains above the RPE) (Kim et al., 2018). It is not clear however why PCV, similarly located underneath the RPE, is associated with the least occurrence of prechoroidal clefts, although this may be due to different adhesions or other mechanisms of fluid leakage.

The influence of anti‐VEGF therapy on prechoroidal cleft development is unclear, with theories that either promote development or regression. It was observed that some prechoroidal clefts resolved after more frequent injections or a switch to other types of anti‐VEGF medication (Rahimy et al., 2014), which could be explained as decreased fluid activity. On the other hand, anti‐VEGF also potentially increases the contractile forces which could cause prechoroidal cleft enlargement. Mostly however, the prechoroidal cleft size decreases following start of anti‐VEGF therapy (Cozzi et al., 2022; Kim et al., 2022).

Apart from the earlier two driving forces of prechoroidal cleft development, there may be a component of dysfunctional RPE, which would explain the higher presence of SRF in eyes with an prechoroidal cleft (Kredi et al., 2023). It may also be that different factors contribute based on timing of the cleft development, by the hypothesis that late‐occurring clefts relate to more chronic alterations and subfoveal fibrosis (Kim et al., 2017).

### 3.7 Future research

Current knowledge on the prechoroidal cleft is derived from observational studies using imaging techniques only. A gold standard work would be to study the (retinal and choroidal) tissue and presumed fluids of eyes with a prechoroidal cleft using histology and laboratory analysis. Histologic studies may explain the composition of the sub‐RPE material with presumed contractile properties and may elaborate on the adhesions between Bruch's membrane and the RPE to explain the outward bowing as is seen in the cleft. Analysis of the presumed fluid in the cleft might help to find its origin. These suggested investigations would impose practical and ethical difficulties however, since it requires invasive biopsies or surgical removal of the eye without direct benefit for the individual patient. Cytokine analysis of vitreous or anterior chamber fluid may be more feasible as a first step, to study differences in vascular growth factors and inflammatory mediators in eyes developing a prechoroidal cleft. This may elucidate the relation with nAMD disease activity, though it may be only indirectly related to the cleft itself.

The behaviour of prechoroidal clefts over time is not fully understood, and neither is the natural history in absence of ocular treatment. Current studies that report on the cleft, include cohorts of patients who receive various types of anti‐VEGF or PDT laser treatments (for the associated nAMD or PCV), and the influence of this on the cleft itself is not known. It may be interesting to study clefts over time in non‐treated nAMD eyes (e.g. lacking therapy because of low visual acuity) to study the natural history. There is currently no direct support that anti‐VEGF treatment has an influence on the size of a prechoroidal cleft, though it is observed that clefts commonly resolve in eyes that concurrently receive anti‐VEGF treatment. Work on its aetiology may therefore be needed to identify targets for treatment. Even then, however, it needs to be studied if treatment of the cleft is beneficial for the functional outcome of the patient.

Findings regarding the incidence of RPE tears and subretinal haemorrhages in eyes with a prechoroidal cleft are currently conflicting, although it is suggested that eyes with a prechoroidal cleft are at higher risk of developing these complications. Larger, systematically controlled studies are needed to find the relevance of this suggested observation.

We observe that the prechoroidal cleft has, to our knowledge, not been reported in diseases other than nAMD. This warrants further investigation as the condition may be overlooked. If indeed more prevalent in nAMD, it would be interesting to study why nAMD is associated with prechoroidal cleft development, and other MNV‐related diseases are not.

A final remark on the prechoroidal cleft is semantic, as its description may appear to be overlapping with certain types of fibrovascular RPE detachments. As noted before, the outward bowing of Bruch's membrane, and a more‐or‐less lenticular‐shaped hyporeflective space, is a distinctive feature that should differentiate a prechoroidal cleft from a ‘regular’ fibrovascular RPE detachment. This may be difficult to delineate in practice from a mixed serous‐fibrovascular RPE detachment. The question however would be if there is a functional difference between different collections of sub‐RPE fluid, and it may be that these entities are a presentation of the same phenomenon in a varying scale. More research is needed to describe these features and to define the proper ocular conditions.

## 4 CONCLUSION AND RECOMMENDATIONS

The prechoroidal cleft is a commonly occurring feature in eyes with nAMD, found in 8–22% of patients, most often associated with MNV type 1 and 3, and less often with MNV type 2 or PCV. The presence of a prechoroidal cleft is associated with poor visual prognosis, and some studies report more associated RPE tears and subretinal haemorrhages, though the literature is not conclusive on this finding. Eyes with a prechoroidal cleft require more frequent anti‐VEGF injections to treat the nAMD and more often require a switch to other types of anti‐VEGF medication compared to eyes without a prechoroidal cleft.

In clinical practice, the prechoroidal cleft should be recognized and differentiated from other (subretinal) fluid or even choroidal lesions. This is important as SRF requires treatment, and choroidal lesions require further diagnostic procedures (as e.g. ultrasound), while the prechoroidal cleft itself requires none of this. Because of its unfavourable prognostic association, clinicians should be even more cautious in patients with a prechoroidal cleft, to provide optimal treatment for the related nAMD. Further work is needed to elucidate the pathophysiology of the prechoroidal cleft and its exact role as a biomarker in nAMD.

## AUTHOR CONTRIBUTIONS

**Niels J. Brouwer:** Conceptualization, methodology, investigation, writing—original draft, writing—review and editing. **T. H. Khanh Vu:** Conceptualization, writing—review and editing. **Yvonne De Hesse‐Jong:** Conceptualization, writing—review and editing. **Elon H. C. van Dijk:** Conceptualization, writing—review and editing, supervision.

## FUNDING INFORMATION

This research did not receive any specific funding.

## CONFLICT OF INTEREST STATEMENT

The authors have no conflict of interest to disclose.

## Supporting information


**Figure S1.** PRISMA style flow chart of the literature search.

## References

[aos70035-bib-0001] Cozzi, M. , Monteduro, D. , Parrulli, S. , Ristoldo, F. , Corvi, F. , Zicarelli, F. et al. (2022) Prechoroidal cleft thickness correlates with disease activity in neovascular age‐related macular degeneration. Graefe's Archive for Clinical and Experimental Ophthalmology, 260, 781–789. 10.1007/s00417-021-05384-w PMC8850287 34491426

[aos70035-bib-0002] Fleckenstein, M. , Schmitz‐Valckenberg, S. & Chakravarthy, U. (2024) Age‐related macular degeneration: a review. JAMA, 331, 147–157. 38193957 10.1001/jama.2023.26074 PMC12935482

[aos70035-bib-0003] Forte, P. , Ferro Desideri, L. , Manocchio, R. , Corazza, P. , Traverso, C.E. & Nicolò, M. (2024) Prechoroidal cleft regression after switch to Intravitreal brolucizumab. European Journal of Ophthalmology, 34, Np123–Np126. 37415410 10.1177/11206721231185903

[aos70035-bib-0004] Hayashi‐Mercado, R. , Pérez‐Montaño, C. , Reyes‐Sánchez, J. & Ramírez‐Estudillo, A. (2022) Findings of uncertain significance by optical coherence tomography (OCT) as prognostic factors in neovascular age‐related macular degeneration (nAMD) treated with ranibizumab. International Journal of Retina and Vitreous, 8, 29. 35449032 10.1186/s40942-022-00379-z PMC9022246

[aos70035-bib-0005] Khan, S. , Engelbert, M. , Imamura, Y. & Freund, K.B. (2012) Polypoidal choroidal vasculopathy: simultaneous indocyanine green angiography and eye‐tracked spectral domain optical coherence tomography findings. Retina, 32, 1057–1068. 22127224 10.1097/IAE.0b013e31823beb14

[aos70035-bib-0006] Kim, I. , Ryu, G. & Sagong, M. (2022) Morphological features and prognostic significance of multilayered pigment epithelium detachment in age‐related macular degeneration. The British Journal of Ophthalmology, 106, 1073–1078. 33658232 10.1136/bjophthalmol-2020-318616

[aos70035-bib-0007] Kim, J.H. , Chang, Y.S. , Kim, J.W. , Kim, C.G. & Lee, D.W. (2018) Prechoroidal cleft in type 3 neovascularization: incidence, timing, and its association with visual outcome. Journal of Ophthalmology, 2018, 2578349. 30581602 10.1155/2018/2578349 PMC6276463

[aos70035-bib-0008] Kim, J.H. , Kim, J.W. , Kim, C.G. & Lee, D.W. (2021) Long‐term course and visual outcomes of Prechoroidal cleft in Neovascular age‐related macular degeneration and Polypoidal choroidal vasculopathy. Retina, 41, 2436–2445. 34173365 10.1097/IAE.0000000000003242

[aos70035-bib-0009] Kim, J.M. , Kang, S.W. , Son, D.Y. & Bae, K. (2017) Risk factors and clinical significance of Prechoroidal cleft in Neovascular age‐related macular degeneration. Retina, 37, 2047–2055. 28114175 10.1097/IAE.0000000000001435

[aos70035-bib-0010] Kredi, G. , Iglicki, M. , Gomel, N. , Hilely, A. , Loewenstein, A. , Habot‐Wilner, Z. et al. (2023) Risk factors and clinical significance of prechoroidal cleft in eyes with neovascular age‐related macular degeneration in Caucasian patients. Acta Ophthalmologica, 101, e338–e345. 36259092 10.1111/aos.15273

[aos70035-bib-0011] Mrejen, S. , Sarraf, D. , Mukkamala, S.K. & Freund, K.B. (2013) Multimodal imaging of pigment epithelial detachment: a guide to evaluation. Retina, 33, 1735–1762. 23873168 10.1097/IAE.0b013e3182993f66

[aos70035-bib-0012] Mukai, R. , Sato, T. & Kishi, S. (2014) A hyporeflective space between hyperreflective materials in pigment epithelial detachment and Bruch's membrane in neovascular age‐related macular degeneration. BMC Ophthalmology, 14, 159. 25515712 10.1186/1471-2415-14-159 PMC4274686

[aos70035-bib-0013] Nagiel, A. , Freund, K.B. , Spaide, R.F. , Munch, I.C. , Larsen, M. & Sarraf, D. (2013) Mechanism of retinal pigment epithelium tear formation following intravitreal anti‐vascular endothelial growth factor therapy revealed by spectral‐domain optical coherence tomography. American Journal of Ophthalmology, 156, 981–988.e982. 23972309 10.1016/j.ajo.2013.06.024

[aos70035-bib-0014] Pece, A. , Borrelli, E. , Sacconi, R. , Maione, G. , Bandello, F. & Querques, G. (2019) Choroidal cleft simulating choroidal caverns in neovascular age‐related macular degeneration. European Journal of Ophthalmology, 29, 471–473. 31353948 10.1177/1120672119855540

[aos70035-bib-0015] Rahimy, E. , Freund, K.B. , Larsen, M. , Spaide, R.F. , Costa, R.A. , Hoang, Q. et al. (2014) Multilayered pigment epithelial detachment in neovascular age‐related macular degeneration. Retina, 34, 1289–1295. 24675391 10.1097/IAE.0000000000000130

[aos70035-bib-0016] Sariyeva Ismayilov, A. , Duru, Y. , Çağlar, T. , Erseven, C. & Ulusoy, M.O. (2024) Predictive factors of age‐related macular degeneration with poor response to three loading doses of anti‐vascular endothelial growth factor. International Ophthalmology, 44, 264. 38913217 10.1007/s10792-024-03198-3

[aos70035-bib-0017] Sirks, M.J. , van Dijk, E.H.C. , Ghalayini, H. et al. (2025) The clinical spectrum of polypoidal choroidal vasculopathy in Caucasian patients: a retrospective multicenter cohort study. Ophthalmology Retina, 44, 122. 10.1016/j.oret.2025.04.019 40316047

[aos70035-bib-0018] Spaide, R.F. , Jaffe, G.J. , Sarraf, D. , Freund, K.B. , Sadda, S.R. , Staurenghi, G. et al. (2020) Consensus nomenclature for reporting neovascular age‐related macular degeneration data: consensus on neovascular age‐related macular degeneration nomenclature study group. Ophthalmology, 127, 616–636. 31864668 10.1016/j.ophtha.2019.11.004 PMC11559632

[aos70035-bib-0019] Tenbrock, L. , Wolf, J. , Boneva, S. , Schlecht, A. , Agostini, H. , Wieghofer, P. et al. (2022) Subretinal fibrosis in neovascular age‐related macular degeneration: current concepts, therapeutic avenues, and future perspectives. Cell and Tissue Research, 387, 361–375. 34477966 10.1007/s00441-021-03514-8 PMC8975778

[aos70035-bib-0020] Yeom, H. , Kwon, H.J. , Kim, Y.J. , Lee, J. , Yoon, Y.H. & Lee, J.Y. (2023) Real‐world study to evaluate the efficacy and safety of intravitreal brolucizumab for refractory neovascular age‐related macular degeneration. Scientific Reports, 13, 11400. 37452068 10.1038/s41598-023-38173-y PMC10349130

