# Supplementary material for: The prechoroidal cleft in neovascular age‐related macular degeneration
Source: Acta Ophthalmol. 2025 Nov 11;104(4):364–75. doi: 10.1111/aos.70035 (PMC13166397; doi:10.1111/aos.70035)
Supplement: Supplementary file 1 — Figure S1. PRISMA style flow chart of the literature search. [file AOS-104-364-s001.pdf]

Supporting Figure S1. PRISMA-style flow chart of the literature search

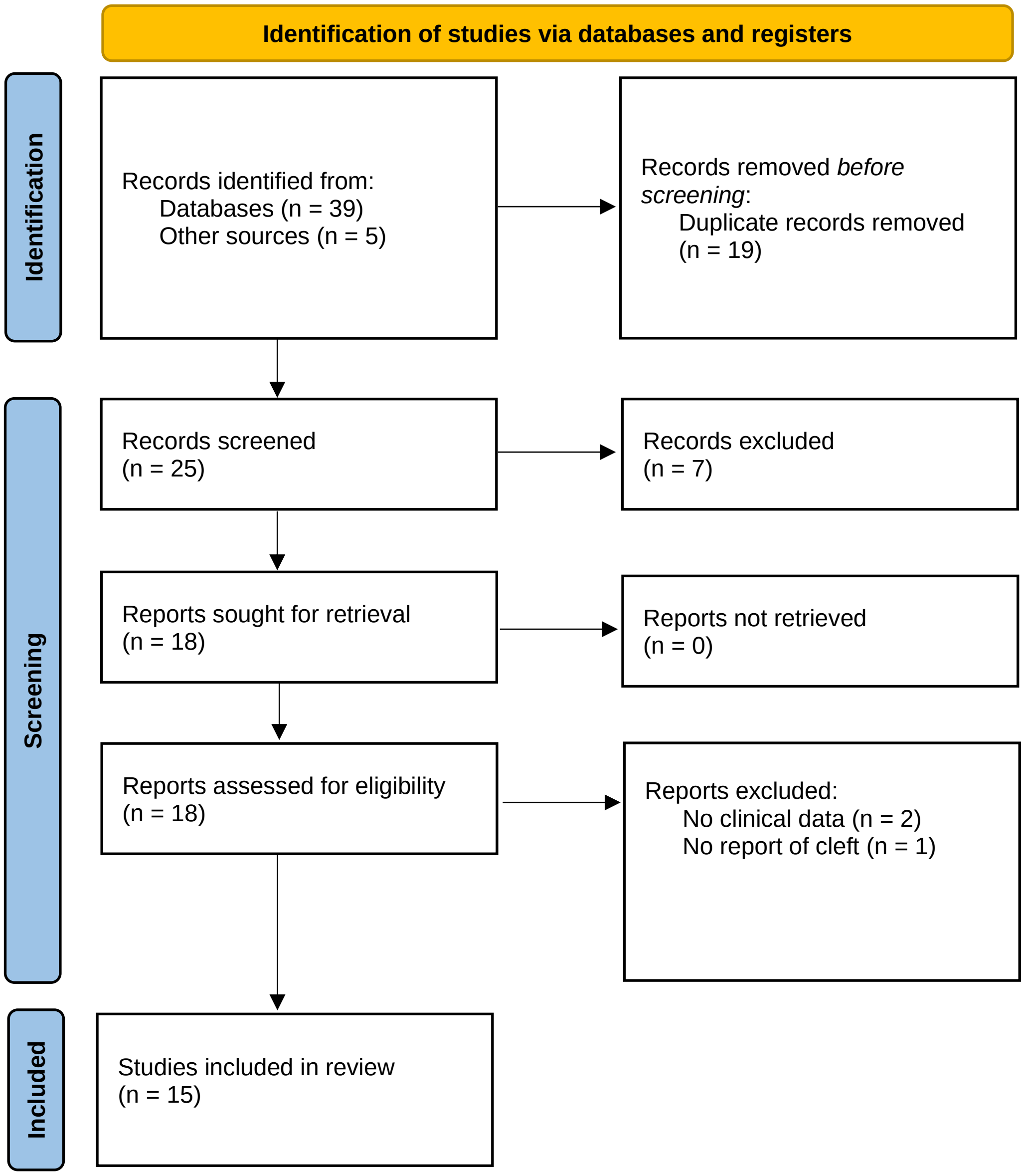

Source: Page MJ, McKenzie JE, Bossuyt PM, et al. (2021): The PRISMA 2020 statement: an updated guideline for reporting systematic reviews. *BMJ*, 372; n71
